# Using Mobile Health Gamification to Facilitate Cognitive Behavioral Therapy Skills Practice in Child Anxiety Treatment: Open Clinical Trial

**DOI:** 10.2196/games.8902

**Published:** 2018-05-10

**Authors:** Gede Pramana, Bambang Parmanto, James Lomas, Oliver Lindhiem, Philip C Kendall, Jennifer Silk

**Affiliations:** ^1^ Department of Health Information Management University of Pittsburgh Pittsburgh, PA United States; ^2^ The Design Lab University of California San Diego, CA United States; ^3^ Department of Psychiatry University of Pittsburgh Pittsburgh, PA United States; ^4^ Department of Psychology Temple University Philadelphia, PA United States; ^5^ Department of Psychology University of Pittsburgh Pittsburgh, PA United States

**Keywords:** gamification, mobile health, ecological momentary intervention, cognitive behavioral therapy, child anxiety treatment, SmartCAT, childhood anxiety disorders

## Abstract

**Background:**

Cognitive behavioral therapy is an efficacious treatment for child anxiety disorders. Although efficacious, many children (40%-50%) do not show a significant reduction in symptoms or full recovery from primary anxiety diagnoses. One possibility is that they are unwilling to learn and practice cognitive behavioral therapy skills beyond therapy sessions. This can occur for a variety of reasons, including a lack of motivation, forgetfulness, and a lack of cognitive behavioral therapy skills understanding. Mobile health (mHealth) gamification provides a potential solution to improve cognitive behavioral therapy efficacy by delivering more engaging and interactive strategies to facilitate cognitive behavioral therapy skills practice in everyday lives (in vivo).

**Objective:**

The goal of this project was to redesign an existing mHealth system called SmartCAT (Smartphone-enhanced Child Anxiety Treatment) so as to increase user engagement, retention, and learning facilitation by integrating gamification techniques and interactive features. Furthermore, this project assessed the effectiveness of gamification in improving user engagement and retention throughout posttreatment.

**Methods:**

We redesigned and implemented the SmartCAT system consisting of a smartphone app for children and an integrated clinician portal. The gamified app contains (1) a series of interactive games and activities to reinforce skill understanding, (2) an in vivo skills coach that cues the participant to use cognitive behavioral therapy skills during real-world emotional experiences, (3) a home challenge module to encourage home-based exposure tasks, (4) a digital reward system that contains digital points and trophies, and (5) a therapist-patient messaging interface. Therapists used a secure Web-based portal connected to the app to set up required activities for each session, receive or send messages, manage participant rewards and challenges, and view data and figures summarizing the app usage. The system was implemented as an adjunctive component to brief cognitive behavioral therapy in an open clinical trial. To evaluate the effectiveness of gamification, we compared the app usage data at posttreatment with the earlier version of SmartCAT without gamification.

**Results:**

Gamified SmartCAT was used frequently throughout treatment. On average, patients spent 35.59 min on the app (SD 64.18) completing 13.00 activities between each therapy session (SD 12.61). At the 0.10 significance level, the app usage of the gamified system (median 68.00) was higher than that of the earlier, nongamified SmartCAT version (median 37.00, *U*=76.00, *P*<.01). The amount of time spent on the gamified system (median 173.15) was significantly different from that of the earlier version (median 120.73, *U*=173.00, *P*=.06).

**Conclusions:**

The gamified system showed good acceptability, usefulness, and engagement among anxious children receiving brief cognitive behavioral therapy treatment. Integrating an mHealth gamification platform within treatment for anxious children seems to increase involvement in shorter treatment. Further study is needed to evaluate increase in involvement in full-length treatment.

## Introduction

### Background

The results of multiple independent randomized clinical trials provide evidence that cognitive behavioral therapy (CBT) is an efficacious treatment for childhood anxiety [1-7]. CBT typically requires 10 to 20 weeks of weekly sessions with a CBT therapist [8] and emphasizes the importance of CBT skills rehearsal, exposure tasks, and practice beyond office visits (homework) [9-13]. Markedly, about 40% of anxious children receiving CBT treatment show little recovery from primary anxiety diagnoses, despite evidence suggesting CBT is an efficacious treatment [8]. One possibility is that treatment requires a willingness to learn and practice CBT skills beyond therapy sessions.

Although homework is routinely assigned, many anxious children struggle with homework completion [14] possibly because of a lack of therapeutic commitment or motivation [15]. Unlike adults who are often self-referred, children are usually brought to therapy by their parents or caregivers. As a result, these children are not always considered to be “voluntary participants” in therapy and may view homework as unfavorable [16]. Therapists note that other noncompliance factors include forgetfulness and lack of understanding of CBT skills [14].

### Overcoming Barriers to Home-Based Skills Practice for Children

Mobile health (mHealth) technologies present potential solutions to overcoming barriers to fostering home-based skills practice for children. First, the “always-carried” and “always-on” nature of smartphones creates an opportunity to deliver CBT interventions to children in natural settings during their everyday lives, an approach referred to as “ecological momentary intervention” (EMI) [17]. EMIs can provide skills coaching to anxious children in the real world outside of sessions, when it is most needed. Anxious children can also access training materials *in situ* at their convenience throughout the day. Second, the increased processing and sensing capability of smartphones allows for more sophisticated, interactive, and engaging health intervention apps. This provides an opportunity for developers to make context-aware mHealth apps that can automatically detect when and where children require skills coaching during real-world emotional situations.

Despite this potential, the repetitive tasks (eg, self-monitoring and self-management) that characterize most mHealth apps can be exhausting and may lack intrinsic rewards [18]. An alternative to traditional mHealth apps is the use of gamification, one of many persuasive approaches that uses game design elements to engage people in nongame contexts [19]. Among children, the use of gamification is particularly effective in addressing the problem of lack of motivation [20]. When integrated with mHealth apps, gamification can potentially make tedious activities on mHealth apps more engaging to children, thus increasing their motivation to use them.

### Goal of This Project

The purpose of this project was multifaceted. First, we redesigned our existing mHealth system, titled “Smartphone-enhanced Child Anxiety Treatment” (SmartCAT), consisting of a smartphone app (SmartCAT app), a therapist portal (SmartCAT portal), and a two-way communication connecting them [21]. This redesign included gamification techniques as well as a number of interactive skill-builder modules to increase user engagement or retention and facilitate learning. Second, we evaluated the utility of the redesigned SmartCAT as an adjunctive component to CBT treatment in an open clinical trial. User engagement data (ie, time spent on app and app use) and the app retention (app use per session) at posttreatment were used to assess the utility. Finally, the effectiveness of gamification was evaluated by comparing user engagement data with the previous version of SmartCAT without gamification.

## Methods

### User-Centered Design

A user-centered design (UCD) approach was used to gather requirements and iteratively design the system, leveraging the SmartCAT 1.0 system that had been previously pilot-tested [21]. In this version, the app notifies patients to initiate a “skills coach” module, which then cues patients to complete a series of questions about recent emotional events and to apply skills learned in therapy toward coping with that event. Throughout this study, the skills coach was scheduled from the portal by the therapist to launch automatically once per day (either at a fixed or random time depending on patients’ desire) and be completed more frequently if desired by the patient. It could also be activated at “opportune” moments when patients were experiencing acute anxiety. After completing a skills coach entry, patients were rewarded with digital points that could be “cashed in” for a prize.

Although the skills coach in SmartCAT 1.0 was actively used, averaging 5.36 times per session, children and therapists suggested several potential improvements including developing more interactive and fun ways for the children to learn and practice CBT skills in daily life and also improving rewards to increase the rates of CBT skills practice. On the basis of this feedback, we redesigned and developed a new system using the iterative, user-centered approach described below.

The SmartCAT addresses barriers to home-based skills practice for children by (1) Providing automatic cues to children to practice skills at prescribed times and places, even when they forget to initiate skills practice on their own; (2) Motivating children to practice skills; and (3) Providing interactive ways to learn the skills and offering *in situ* learning exercises to increase understanding of skills as well as daily personalized home-based exposures. To achieve this goal, a UCD approach was used to identify key components of the system by involving CBT therapists and children in the process. Because it emphasizes user perspective or context, UCD is an appropriate method for achieving a balance between fun and function (in formal terms, between the internal goal of the system and the treatment goal of improving skills understanding) [22].

The UCD process was conducted in three steps. The initial step of this UCD process was the development of design principles based on user information captured and interpreted by therapists that deliver CBT to anxious children. The therapists served as the interface between the users (ie, anxious children) and the designer or software developer. Meetings with therapists were conducted to brainstorm and identify design ideas and criteria. Such ideas as including interactive features, treatment engagement and adherence, and educational content were addressed. These design ideas and criteria were then translated into design principles, which were in turn used to evaluate the system. The results from the design principles development step provided general guidelines for implementation by software developers for the iterative system development step. Continuous input or feedback was provided by two therapists during the system development process. A formative usability study involving the children in the study was conducted following the system development process to collect feedback and discover usability problems. Revisions were made before the system was implemented as part of the clinical trial.

The initial UCD process revealed a conceptual model for the system that includes seven key components. By implementing these components in the system, CBT treatment outcomes can hopefully be improved. The individual and specific components of the system are outlined as follows:

1. *Reminder*: home-based skills practice is often less impactful because of children forgetting to practice CBT skills beyond the clinic. According to behavioral learning theory, behavior depends on internal (thoughts) or external (environmental) stimuli or cues [23]. This means that noncompliant behaviors such as not remembering to practice CBT skills can be modified by introducing repetition of external stimuli or cues such as reminders*.*
2. *Game- or multimedia-based coping skills learning*: CBT for anxious children aims at reducing anxiety and preventing relapse. As CBT is a skills-based treatment, much of the work associated with treatment involves teaching children new behaviors, concrete problem-solving skills, and strategies for challenging anxious thoughts and beliefs. To improve the learning process, game-based learning can be used as an appropriate way to provide an interactive yet fun learning environment beyond the weekly in-person sessions. Ultimately, game-based learning provides a type of game play that has well-defined learning outcomes. Moreover, research suggests that games can effectively model the learning process in that games require players to be active and to provide immediate feedback as a result of players’ decisions during game play [24]. To facilitate the learning of coping skills, several CBT components—such as emotion and somatic symptoms identification, cognitive restructuring, and problem solving—were translated into game formats. Audio or video recording were also used to improve such coping exercises such as deep breathing or relaxation.ing, and problem-solving was translated into game format.nts such as emotion and somatic symptoms identification, cognitive rest
3. *Step-based plan for dealing with anxiety*: the “Coping Cat” program is a structured CBT program that was developed at Temple University’s Child and Adolescent Anxiety Disorders Clinic [25,26]. Notably, CBT skills training is one of the two key components of the Coping Cat program. Here, anxious children learn several basic skills that are then integrated into a plan for dealing with anxiety called the FEAR plan. The FEAR plan comprises four concepts addressed in the following, easily remembered anagram: (1) *Feeling frightened?* This step aims to increase awareness of physical symptoms of anxiety; (2) *Expecting bad things to happen?* Here, the focus is on recognizing anxious self-talk; (3) *Attitudes and actions that will help*. In this step, participants develop behavior and coping talk to use when anxious; and (4) Results and rewards. This final step comprises a self-evaluation and administration of reward for effort.
4. *Exposure tasks*: another important component of CBT is skills practice, which involves having the children experience anxious distress in real anxiety-provoking situations. Exposure tasks tailored to the children’s fears are conducted once the children demonstrate an understanding of the concept within the FEAR plan (based on the therapist’s clinical judgment). To facilitate exposure task practice, a list of in vivo tasks that the children need to conduct were included. The therapist collaborates with the children to prepare the list [27].
5. *Therapist-patient interaction*: to support therapist-patient interaction beyond office visits, a Health Insurance Portability and Accountability Act–compliant messaging system is required. Using this feature, a participant can compose a message on his or her phone, and the message will be sent to a Web-based portal rather than the therapist’s private phone. This protects the private space of the clinician and allows the communication to be part of the treatment record.
6. *Reinforcement through gamification*: one way to improve homework compliance is by providing positive reinforcement in the form of rewards (eg, small toys, accessories or makeup, and gift cards) for completing homework [16,28]. In many manual-based CBT treatment (eg, the Coping Cat program), therapists acknowledge or praise participants’ efforts to engage in exposure challenges (eg, talking with 5 people) by rewarding them with collectible cards (eg, baseball card), stickers, or small toys. Gamification techniques, which add gameful (rule-based and goal-oriented) experience, can provide positive reinforcement to anxious children by rewarding their efforts in completing homework.
7. *Usage monitoring*: as part of clinician-directed CBT treatment, the therapists are required to monitor a participant’s adherence to treatment regimens and activities. The therapist can then use the monitoring data to determine the treatment regimen for the upcoming week.

The CBT components of the model were translated into several skill-builder modules that include an in vivo skills coach, a series of interactive games and activities to reinforce skill understanding, and a home challenge module to encourage home-based exposure (Table 1). Other skill-building activities such as viewing or practicing with a deep breathing techniques video, listening or practicing with a progressive muscle relaxation audio file, or practicing a weekly task adapted from the Coping Cat workbook were provided. The number and types of skill-builder modules can be adjusted in accordance with the children’s progress during CBT treatment.

### Implementation of Gamification

Gamification aims to increase people’s engagements in real life activities and encourage specific human behaviors. To some extent, the concept is already being used in manual-based CBT treatment such as the Coping Cat program [25]. During weekly sessions, eg, therapists acknowledge or praise children’s efforts to engage in exposures challenges (eg, talking with 5 people) by rewarding them with collectible cards (eg, baseball card), stickers, or small toys.

Recent advances in interactive mHealth technologies allow gamification concepts to be layered on top of activities provided by mobile apps. “Swarm” app, eg, rewards its users for checking into a new place by giving digital coins, badges, stickers, and statuses. These game mechanics serve dual functions—helping users learn to use the app and making a real-world experience more engaging. Digital coins and badges give the users a sense of accomplishment, whereas status changes such as “mayorships” allow users to compete with their friends.

In this project, the system was gamified so as to drive children’s engagement in completing their weekly skill-builder modules via an iterative process consisting of four steps. These steps are as follows:

1. Identify the end goals**:** identify the desired goals (ie, desired human behaviors). When defining goals, the contexts of implementation (eg, education and health) and the needs or requirements imposed by stakeholders (eg, a policy of screen time reduction for children and smartphone use in class) must be considered [29]. Ideally, the goals should be specific (clear and well-defined), measurable, attainable, and intended to support and enhance the existing context. In this project, the goal was to maintain participants’ therapeutic commitment or motivation in completing between-sessions skill-builder activities.
2. Determine interesting activities to move patients toward the end goals: identify activities that are aligned with the goals. The activities should also capture the interest of the person. From a self-determination theory perspective, interest can be defined as an affect that occurs in the interaction between a person and an activity [30]. Interest organizes people’s attention and activity. When people experience interest (being intrinsically motivated), the energy necessary for action is readily available because they are rewarded with spontaneous affective or cognitive experiences accompanying their behavior. Ryan and Deci [31] explain that intrinsic motivation can be maintained by satisfying three psychological needs:
  - Competence: the need of people to gain mastery of tasks and learn different skills. When people feel that they have skill or expertise at doing something, they will be more likely to continue doing it. Opportunities to learn different skills, or be optimally challenged, can also improve a person’s level of competency [32].
  - Autonomy: refers to the need to feel in control when performing activities or tasks. The core concept of autonomy is freedom. Allowing individuals freedom in choosing has been shown to improve autonomy and, consequently, their intrinsic motivation [32].
  - Relatedness or connection: refers to the need to feel connected to others. People tend to internalize and accept values and practices from those to whom they feel connected and from contexts in which they experience a sense of belonging. Providing a possibility of social connectedness that conveys security can strengthen intrinsic motivation [33].
  Although interest plays a central role in intrinsic motivation, it is not central to all motivated behavior. People often engage in instrumental activities for some desired outcome not related to the activity itself (being extrinsically motivated). External rewards such as points, money, gift cards, toys, or something tangible can motivate people to complete tasks. For gamification to truly motivate people, it has to target correct and intrinsically motivated activities, as well as provide external rewards for completing the activities [34]. When working with children, extrinsic rewards have been found to be an appropriate form of motivation [35]. Table 2 shows intrinsic and extrinsic motivators that were added to the target activities.
3. Apply game design elements to improve user experience: key elements of gamification are applied to make activities feel more “playful.” Table 3 shows the game design elements that have been implemented. Furthermore, to identify the key elements, we can view game design elements as a hierarchy that contains components, mechanics, and dynamics [36]. “Components” represent the specific forms of mechanics and dynamics. Each component is tied to one or more higher-level elements. “Mechanics” refers to a distinct set of rules or basic processes that generate user engagement and drive the action forward. “Dynamics” represents the big-picture aspects of the gamified system that are indirectly managed by the system. Initially, actions that need monitoring and rewarding are defined. Then, points, badges, and achievements (ie, trophies and stars) are utilized to reward users when performing an action or a collection of actions. Points, levels, badges, and achievements represent the components section of the pyramidal hierarchical structure. To generate engagement, challenges and feedback representing the mechanics section are added. After completing a challenge, the user can collect rewards (ie, tangible payoffs as extrinsic motivators). Ultimately, the dynamics provided by the game element hierarchy system represents the relationship between tangible payoffs and the number of points collected (bigger prizes require one to get a higher number of points).
4. Evaluate effectiveness: depending on the goals defined in the initial step, gathering quantitative or qualitative data can assess the effectiveness of gamification. Quantitative data that includes engagement (time spent using the app, the number of digital points collected) and retention (the number of features completed between sessions) can be used to infer user behavior directly. Qualitative data such as user feedback, comments, concerns, frustrations, and suggestions can capture perceptions and attitudes toward gamified apps.

### Participants

A total of 35 participants (aged 9-14 years; mean=11.19) met the criteria for the fifth edition of the Diagnostic and Statistical Manual of Mental Disorders (DSM-V) diagnosis of generalized anxiety disorder, social anxiety disorder, and/or separation anxiety disorder. These diagnoses are common in children, frequently cooccur, have a similar presentation, and respond to the same treatment approaches [37-39]. A lower age limit of 9 years and an upper limit of 14 years were chosen based on the reading level requirements for the app and the age-appropriateness of the materials, respectively.

The participants included 5 participants enrolled in a beta testing phase and 30 participants enrolled in an open trial phase who completed treatment. The participants who enrolled in the beta testing phase received similar treatment to those who enrolled in the open trial phase.

**Table 1:** Skill-builder modules*.*

Module	Session	Description
Skills coach	1, 2, 3, 4, 5, 6, 7	Guide the participant through developing a FEAR plan for a current or recent in vivo anxious experience.
What’s the feeling? (game)	1, 2, 3, 4^a^, 5^a^, 6^a^, 7^a^	Ask the participant to identify emotional and somatic symptoms from various scenarios (including anxiety, physical pain, and hunger).
Chillax	1, 2^a^, 3^a^, 4^a^, 5^a^, 6^a^, 7^a^	View or practice with a video demonstrating deep breathing techniques.
		Listen or practice with an mp3 audio file for progressive muscle relaxation.
Thought-buster (game)	2, 3^a^, 4, 5, 6, 7	Ask the participant to identify anxious vs nonanxious self-talk or coping vs noncoping self-talk.
Thought-swapper (game)	3, 4, 5, 6, 7	Ask the participant to identify coping self-talk that works best in a given situation.
Problem-solver (game)	3, 4, 5, 6, 7	Generate and evaluate potential solutions to hypothetical problems.
Challenger	4, 5, 6, 7	Therapist selects personally relevant home challenges from a menu on the portal; patient is prompted to develop a FEAR plan and complete these challenges via app.
Show that I can	1, 2, 3, 4, 5, 6, 7	Therapist selects weekly task (adapted from the Coping Cat workbook) from a menu on the portal; patient is prompted to complete the task via app.

^a^Optional.

**Table 2:** Intrinsic and extrinsic motivators in target activities.

Activities	Intrinsic motivators	Extrinsic motivators
Completing interactive skill-building modules (“What’s the feeling?”, Thought-buster, Thought-swapper, Problem-solver)	Specific modules are assigned for a particular session. As the session progresses, different modules with different challenges will be assigned (competence)	Tangible prizes (ie, accessories and makeup, small toys and games, and gift cards for older teens)
	Each module can be initiated independently (autonomy)	
Completing skills coach	As the session progresses, children are asked to come up with their own coping strategies instead of choosing from a provided checklist (competence and autonomy)	
Completing at-home challenges (Challenger), Chillax, and Show that I can task	At-home challenges are discussed with the therapist in face-to-face sessions. Children can choose which challenges they want to complete (competence and autonomy).	
Sending or replying to messages	Children can send messages to their therapist to ask therapeutic questions (relatedness or connection)	Attention, praise

**Table 3:** Actions, components, and mechanics*.*

Actions	Components	Mechanics
Initiate and complete skill-builder modules when requested to do so by app alarm	One point toward the target number of points (cumulative)	Collect a certain number of points. Therapists will assign the target points needed to redeem a selected prize. A collection of stars and a trophy will be displayed on the home screen. A progress bar and badges are displayed after the completion of actions.
Initiate and complete skill-builder modules from within the app (on one’s own initiative).	Two points toward the target number of points (cumulative)	
Complete all required modules for a particular session.	One star	Collect one star for each session.
Complete all required modules for sessions 1, 2, and 3.	Silver trophy	Collect a silver trophy.
Complete all required modules for sessions 4, 5, 6, and 7.	Gold trophy	Collect a gold trophy.

### Procedures

After completing a phone screen, potential participants completed a clinical intake interview. To establish anxiety and exclusionary diagnoses, the Kiddie-Schedule for Affective Disorders and Schizophrenia for School-Aged Children-Present and Lifetime version [40] for DSM-V was used. Participants meeting study criteria were scheduled for a CBT pretest a week before the first therapy session to assess their pretreatment understanding of CBT skills. Each child and a parent or guardian attended an orientation before the first therapy session to learn how to use the smartphone app. Here, the children were provided with an Android smartphone for the duration of the study.

The children were treated using the brief Coping Cat manual and workbook [41,42], implemented over the course of 8 sessions. The treatment includes two key components: (1) CBT skills training, including emotion identification and labeling, cognitive reframing, and problem solving and (2) CBT skills practice through graded exposure to feared stimuli. It should be noted that breathing or muscle relaxation is not formally taught in the brief version. As part of the treatment, the children were asked to complete homework assignments using the app at home. These assignments consisted of specific modules delineated at the end of each session. Treatment was delivered by a master or doctoral level therapist trained in CBT for child anxiety.

As part of the treatment, the therapist was required to complete several tasks via the clinician portal, which is accessible from a computer or a tablet (see Table 4). At the beginning of each session, the therapist, in conjunction with the patient, uses the portal to review the data for the skills coach and other modules from the past week. On the basis of the subsequent discussion and level of patient improvement, the therapist selects germane modules and sets time ranges for the following week. This information is then pushed to the app. To provide motivation and encouragement to the young patient, the therapist integrates immediate rewards (ie, points) into the treatment by managing these rewards directly from the portal. To support clinician-patient interaction, the therapist uses the portal to send or reply to messages to or from patients between sessions. If required, the therapist may also activate the location-aware feature of the app by entering the address of the anxiety-provoking location after discussing it with the patient. In this case, the address is geocoded into a latitude or longitude format by the portal and then sent to the app.

### Measures

#### User Engagement

User engagement was defined as an indicator of the extent to which children interact with the app. User engagement data was reported using indications such as how much time the children spent on the app and the total number of app use during treatment.

#### App Retention

App retention was defined as the extent to which children retain their willingness in completing skill-builder modules between sessions. Retention data was reported using the app use between sessions.

### Statistical Analyses

A Mann-Whitney *U* test was conducted to test whether the gamified system has a higher user engagement rate than the existing version of SmartCAT. A Cronbach alpha level of .10 was used for the test because of the exploratory nature of the study [43]. The Mann-Whitney *U* test was preferred because of an expected nonnormality of the data given the small sample size and possible extreme outliers among participants [44].

**Table 4:** Portal tasks a therapist was required to complete.

Tasks	Start of session	End of session	Between sessions
Enter custom locations and times		✓	
Select modules for upcoming week		✓	
Review skills coach or other module data from the week with child	✓		
Set target points for the following weeks	✓		
Send or receive messages			✓

## Results

### Gamified Mobile Health System (SmartCAT 2.0) for Childhood Anxiety

The app was developed using an Android software development kit (SDK). To accommodate new features (ie, low-power location monitoring and improved user interface), Android SDK version 4.2 or above was used. The minigames were developed using Unity, a cross-platform game engine developed by Unity Technologies. Unity allows the games to be run on top of Android or iPhone operating system (iOS, Apple Inc) devices.

The following key components of the system were implemented during the iterative system development process.

#### Reminder

**Figure 1:**
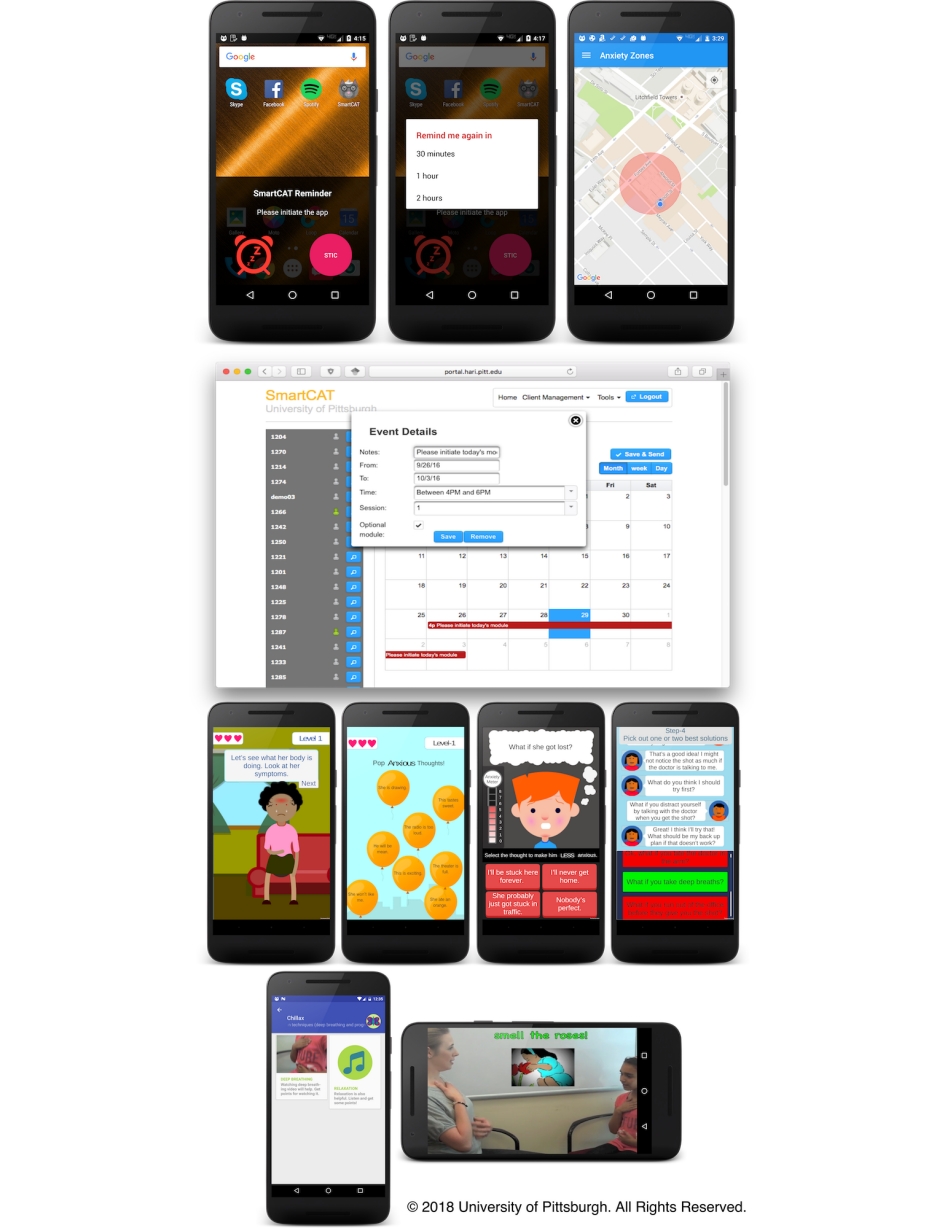
SmartCAT reminders, weekly plan, minigames, and Chillax module screen.

The reminder (Figure 1, line 1) is designed to cue the anxious child toward initiating a skill-builder activity for the day. The app automatically wakes the device, shows a notification dialog, and then plays a distinct sound to get the child’s attention. The dialog contains a customized message, a snooze button, and a shortcut button for initiating the module of the day. If the time is inconvenient, the child can choose to reschedule the reminder later (ie, 30 min, 1 hour, and 2 hours) up to a maximum of three times. To increase the effectiveness of the reminders, the child is also allowed to set their own preprogrammed reminders after completing a skill-builder activity.

To complement time-based reminders, we also provide location-aware reminders using geofencing. Geofencing enables automatic detection of mobile objects as they enter or exit a geofence, which is a virtual boundary for a real-world area [45]. These alert the child, as he or she enter locations that will cause him or her anxiety, to appropriately deal with the situation.

The reminders are integrated into a weekly plan for each child that is pushed to the child’s app. As shown in Figure 1 (line 2), the plan represents a calendar event consisting of four parts:

- Notes: an instructional message that will be shown on the message part of the app’s notification dialog
- Time: the length of the event and the 2-hour window (ie, 4-6 PM, 5-7 PM, 6-8 PM, and 7-9 PM) of the day that a notification should pop up,
- Session: each session is associated with a different set of skill-builder modules
- Optional module: an indicator to include additional skill-builder modules.

#### Game- or Multimedia-Based Coping Skills Learning

The following minigames (see Figure 1, line 3) were developed to provide anxious children more interactive ways to learn important CBT skills such as emotion and somatic symptoms identification, cognitive restructuring, and problem solving.

#### “What’s the feeling?” (Emotion and Somatic Symptoms Identification Skills)

Some anxious children are insufficiently skilled in recognizing somatic cues associated with different feelings (eg, anxiety, anger, boredom, and sadness) [46]. The first thing that the children learn in therapy sessions is how to identify their individual physiological or bodily reactions to anxiety, or more specifically, their own physiological reactions toward anxiety-provoking situations. During the session, the children are shown how physical reactions provide cues associated with anxiety but are also provided with suggestions on how to help their body relax. Moreover, the children learn how to identify and classify what emotions a person is most likely experiencing based on contextual information (eg, scenarios). The “What’s the feeling?” module translates the learning process by asking the child to identify emotional and somatic symptoms from various scenarios.

#### “Thought-Buster” (Cognitive Restructuring Skills)

Clinical levels of anxiety can come from irrational or maladaptive thoughts, beliefs, or self-talk. In therapy sessions, the therapist teaches anxious children cognitive reframing techniques to modify the maladaptive nature of their self-talk. This requires the children to first recognize their self-talk. The “Thought-buster” module helps the child in classifying self-talk as either anxious or nonanxious. Self-talk in the app is presented as balloons that can be popped by tapping the screen and are randomized between screens.

#### “Thought-Swapper” (Cognitive Restructuring Skills)

Rational analysis of thoughts followed by a generation of coping thoughts marks another important task in cognitive restructuring processes. The “Thought-swapper” module guides the child in conducting rational analysis of a thought based on a hypothetical situation. For each hypothetical situation, an anxious thought presents in a thought bubble on top of the character. For each situation, the child needs to either counter the initial thought or intensify it. This way, the child can experiment and learn what coping thoughts will work best in a given situation and foster an understanding that thoughts can influence emotions.

#### Problem-Solver (Problem-Solving Skills)

Anxious children often present with problems they wish to resolve. The strategies (eg, avoidance and escape) these children have used to resolve problems in the past is often not an effective strategy for future difficulties. For example, anxious children might not leave their home to avoid panicky feelings. Although avoidance might be effective in reducing anxious distress in the short term, it is an ineffective strategy for dealing with future uncomfortable thoughts and feelings. During a face-to-face session, a CBT therapist leads the child through the steps in the problem-solving process.

The “Problem-solver” module provides an interactive way for the child to practice the four steps of problem solving: define the problem, come up with as many solutions as you can think of, evaluate all of the options, and pick one or two best solutions. To familiarize the child with these four steps, the module imitates an SMS text message (short message service, SMS) conversation between the child and his or her virtual friend who is experiencing a hypothetical problem from his or her hypothetical life (eg, performing at the talent show after school or going to a friend’s sleepover). Here the child must help his or her virtual friend solve the problem randomly generated each time the module is initiated.

To complement the games, we have included the “Chillax” module (see Figure 1, line 4) that contains a video recording of deep breathing exercises, as well as an audio recording for relaxation. These multimedia files are accessible by initiating the Chillax module—which is part of session-specific skill-builder modules—or by accessing the Media Library.

#### Skills Coach: Step-Based Plan for Dealing With Anxiety

**Figure 2:**
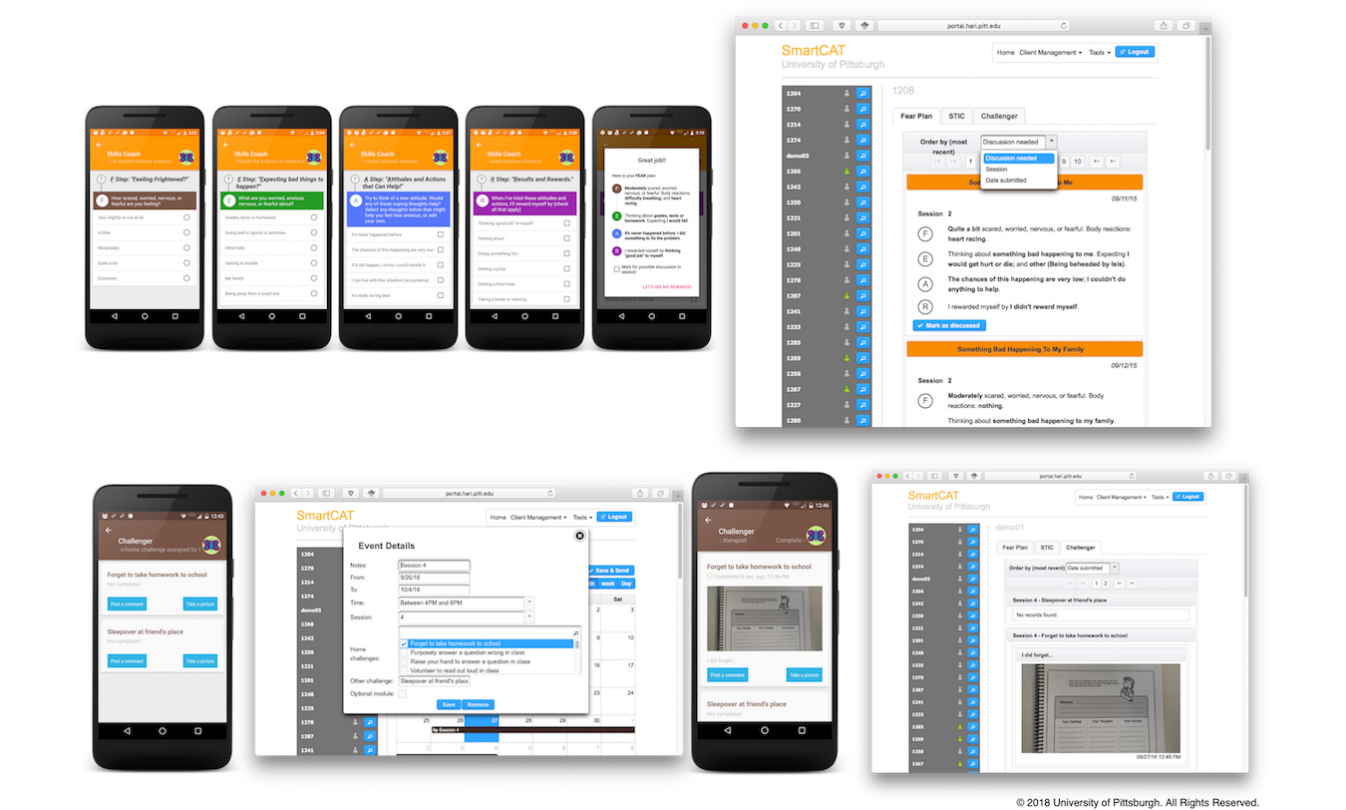
Skills Coach and Challenger module screen.

The “skills coach” module (Figure 2, line 1) provides a series of questions that guide the child in developing a FEAR plan for a current or recent in vivo experience of anxiety. To reduce the child’s burden, checklists are provided. These checklists include common responses to items (ie, typical negative scenarios, automatic thoughts, and coping thoughts) that were generated based on the therapists’ input. As the session advances, the prepopulated responses from the checklist are replaced by text responses that encourage the child to generate his or her own response. FEAR plans are sent to the portal and stored locally on the app for later use when the patient is feeling anxious.

As illustrated in Figure 2, line 1 (right-hand screen), the therapist can review FEAR plans created using the skills coach. The FEAR plans can be ordered by importance (set by the child using the app before FEAR plan submission), session, or submission date. The FEAR plans that need to be discussed with the child have the title appearing over a yellow background.

#### Exposure Tasks

The therapist activates the “Challenger” module (see Figure 2, line 2) from the portal during session four or beyond. This module provides a list of in vivo exposure tasks prepared by the therapist and the child during face-to-face sessions. For each exposure task, the child must describe how each task should be conducted in the “real world situation” and/or provide a photograph showing that he or she completed this task. The child’s response will be sent to the portal for the therapist to see.

#### Therapist-Patient Interaction

**Figure 3:**
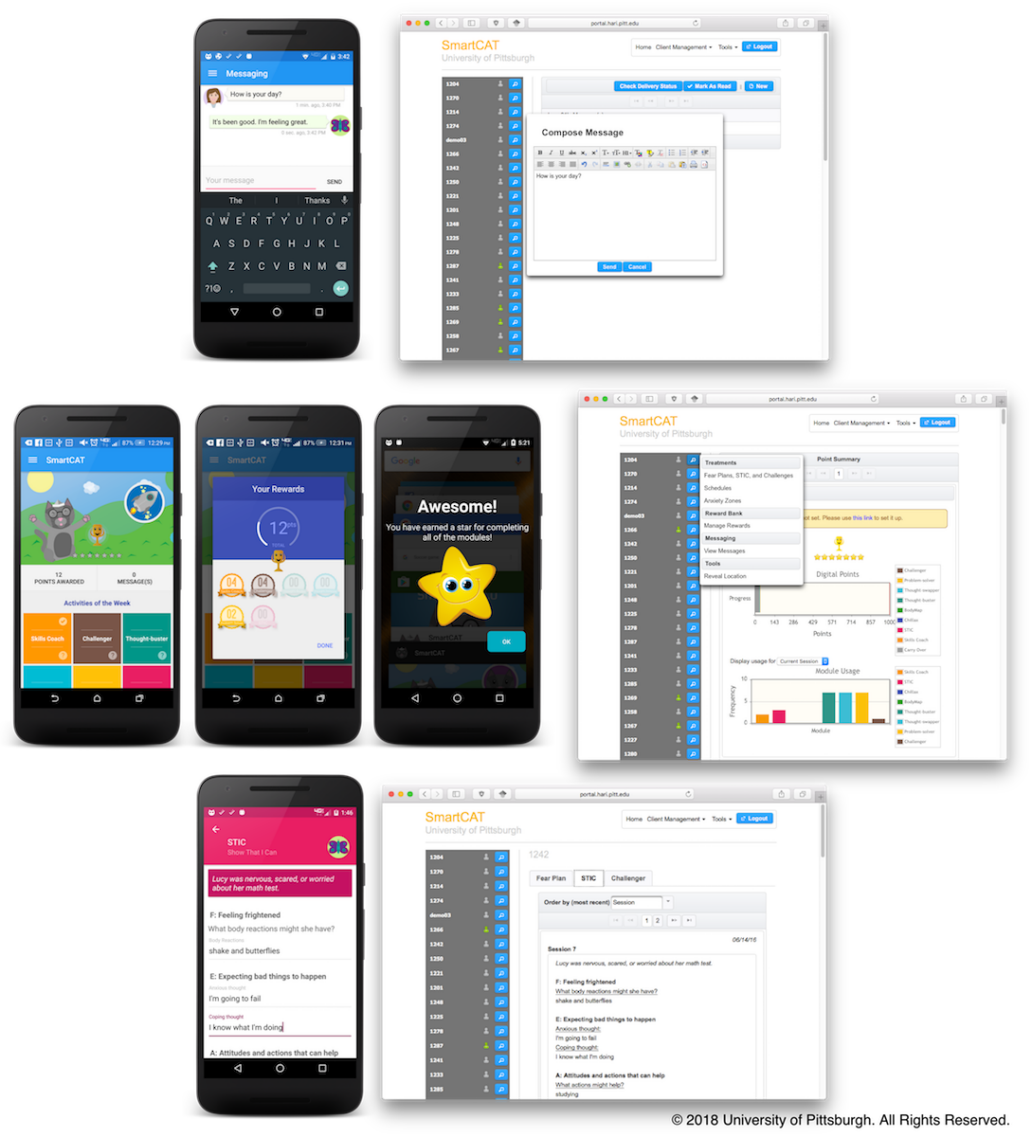
SmartCAT secure messaging system, app and portal home screen, and Show that I can module screen.

To support therapist-patient interaction, we developed a secure messaging interface (Figure 3, line 1). Using this interface, the child can compose a message on the phone, and that message will be sent to the portal rather than the therapist’s private phone. The therapist may view these messages and/or send the child a message at any time using the portal. Incoming or outgoing messages from or to the therapist were encrypted and stored in the phone’s local storage using Advanced Encryption Standard with a 256-bit key. During transmission, these messages were encrypted using Rivest-Shamir-Adleman algorithm with a 2048-bit key to prevent man-in-the-middle attacks. The portal is secure, protected by a corporate firewall.

#### Reinforcement Through Gamification

Skill-builder modules can be activated during instances of acute anxiety by launching the app. From the app’s home screen (Figure 3, line 2), the child can initiate the skill-builder activities that they find most useful. Each time they complete any of the skill-builder modules, digital points are awarded. The target points are associated with a prize that the child can choose and are then assigned by the therapist using the portal. Depending on the target, the points can be redeemed for the desired prize every two or three sessions. If the child acquires digital points beyond the target, however, the remaining digital points will carry over to the following session. A star will be awarded when all of the week’s skill-builder modules are completed. A maximum number of seven stars can be awarded. To maintain patient motivation during treatment, the child is challenged to get a silver trophy for collecting three stars and a gold trophy for collecting the remaining four stars.

#### Usage Monitoring

The portal allows therapists to monitor patients’ progress, as well as access their skills coach, Show that I can, and Challenger entries. The home screen of the portal can be seen illustrated in Figure 3 (line 2; right-hand screen). After successful login, therapists can view a list of their patients and a summary of each patient’s progress. The list provides information about each patient’s smartphone connectivity—a green mark indicating that a patient’s phone is currently connected and a grey mark indicating no connection. An action button, next to the connectivity status, initiates patient-related actions such as reviewing skill-builder module (skills coach, Show that I can, and Challenger) entries, managing treatment regimens and reminders, sending or replying to secure messages, managing geofences, and digital points. A usage summary contains information on the type of trophy and the number of stars collected by each patient. Therapists can also track how far each patient is from the target points and the number and type of skill-builder modules that have been completed.

We have also included the Show that I can module (Figure 3, line 3) that contains session-specific assignments adapted from the Coping Cat workbook. This can be activated by therapists who are utilizing the Coping Cat workbook to provide additional practice with the skills learned in session that week.

### Usage of the Gamified SmartCAT System (SmartCAT 2.0)

**Figure 4:**
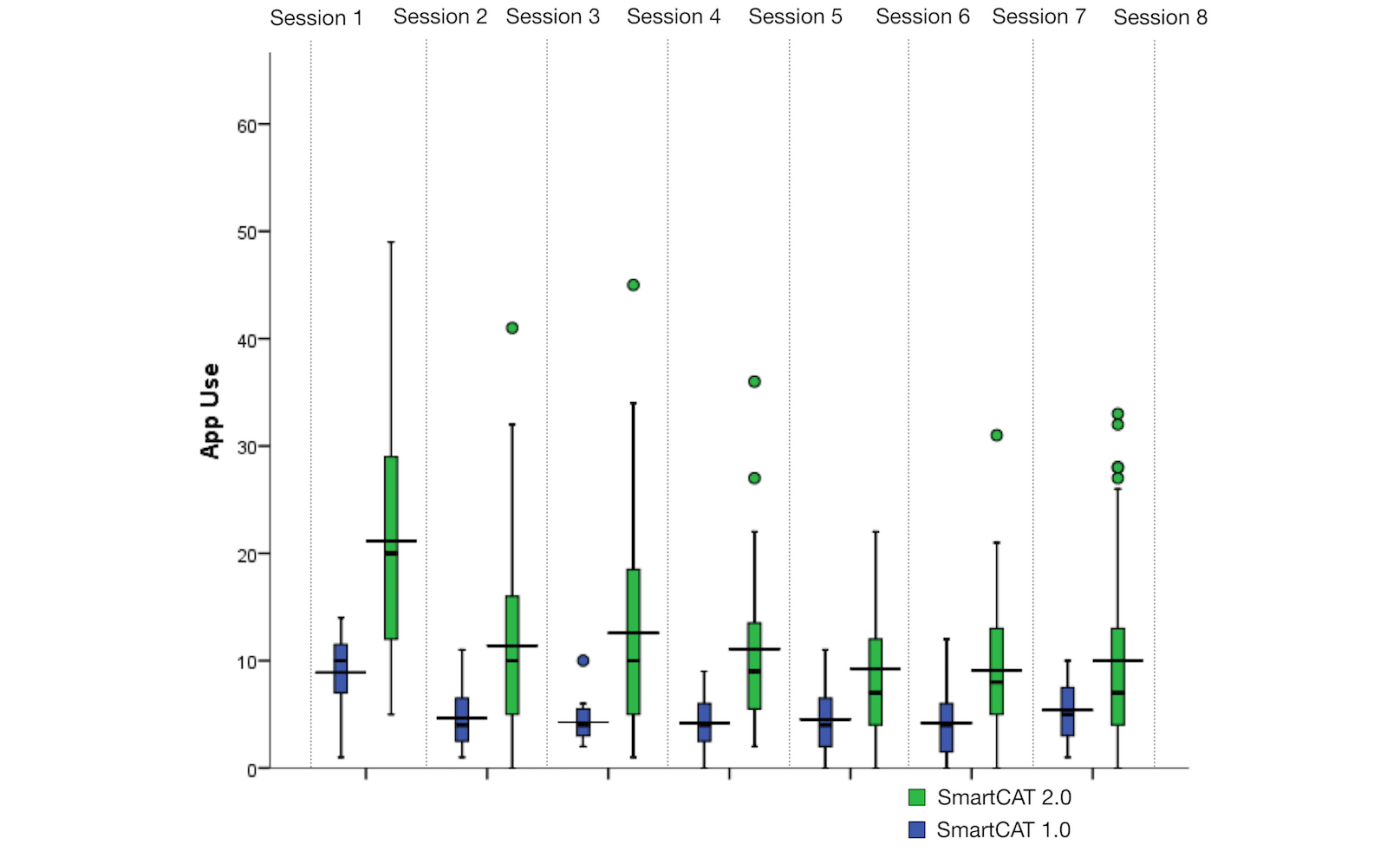
SmartCAT 1.0 vs. SmartCAT 2.0 usage frequency. Usage data were collected after Session 1 and calculated at the end of Session 8.

The child usage data revealed that the app was used frequently during treatment. On average, each child spent 35.59 min on the app (SD 64.18) completing 13.00 skill-builder modules per session (SD 12.61), suggesting high motivation during treatment. Figure 4 shows the app retention of SmartCAT 1.0 compared with that of SmartCAT 2.0. The Y scale represents the app use. The average is represented by the wide horizontal line on each box plot. The median is represented by the short line. App use above 60 modules was considered an outlier and was not included. Although SmartCAT 2.0 was used more often than SmartCAT 1.0 between sessions, the pattern of use between the two systems was arguably consistent. In other words, both systems were highly utilized earlier in the session but then leveled off toward the end.

Table 5 presents the summary of user engagement and retention of the existing and gamified versions, respectively. A two-tailed Mann-Whitney *U* test (Cronbach alpha=.10) indicated that the children were using SmartCAT 2.0 more frequently (median 68.00) than SmartCAT 1.0 (median 37.00, *U*=76.00, *P*<.01), with a large effect size, Cohen *r=*.56. The test also indicated that the children spent longer using SmartCAT 2.0 (median 173.15) than SmartCAT 1.0 (median 120.73, *U*=173.00, *P*=.06), with a medium effect size, Cohen *r*=.27.

**Figure 5:**
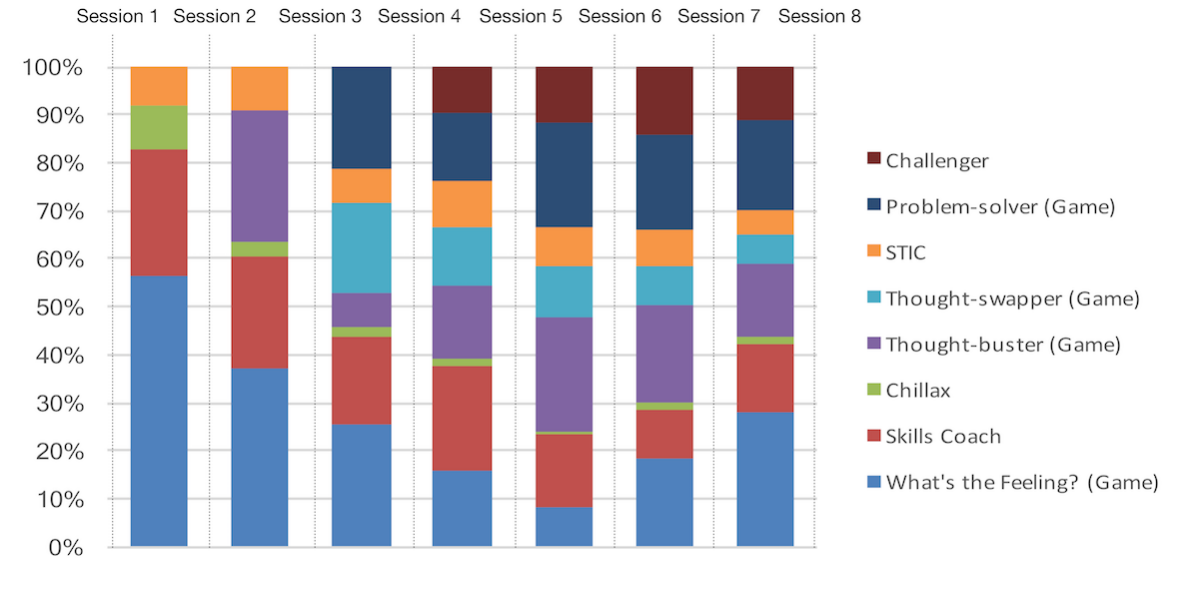
SmartCAT 2.0 module usage between sessions.

The children were using different sets of skill-builder modules between sessions, suggesting their willingness to learn a varying set of skills. As illustrated in Figure 5, the interactive skill-builder modules were completed more frequently than the other modules between sessions. This suggests that the participants were more motivated and likely to engage in learning CBT skills using an interactive and fun learning environment such as games.

**Table 5:** User engagement and app retention by system.

System	Number of participants	Engagement (across duration of treatment)		App retention
		Time spent in minutes (SD)	App use (SD)	App use per session (SD)
SmartCAT 1.0	15	135.08 (56.48)	36.13 (13.54)	5.16 (3.03)
SmartCAT 2.0	35	248.02 (327.41)	90.40 (69.33)	13.00 (12.61)

## Discussion

### Principal Findings

Participants were satisfied with the visual appearance of the app, comfortable using the app, and making the app part of their daily routine. They stated that the app was easy to use and found it helpful when they were experiencing anxiety, as illustrated in the following quotes:

It is amazing, it can really help you.

Patient 1216

I thought the app helped me out a lot. It was like therapist on a phone.

Patient 1240

Was very easy to use and learn. Keep up the good work!

Patient 1302

The app was very easy to use and wasn’t confusing at all.

Patient 1309

On average, the app was used twice a day. The therapists could track participants’ weekly progress and could provide written reinforcements when required using the portal. The result of the implementation indicates that the gamified SmartCAT system has been used as expected and suggests that the inclusion of gamification can effectively increase user engagement and retention.

Although effective, the effects of gamification were not uniformly experienced by all participants. During the clinical trial, one participant did not use the app often, completing only 12 skill-builder modules throughout treatment. This participant was not motivated to use the app and was diagnosed and referred for depression treatment at posttreatment. This suggests that symptoms of depression may interfere with engagement. Six patients used the app more often—but less than an average of seven times—between sessions (<49 times across duration of treatment). This suggests that the implementation of gamification does not always lead to significant increases in user engagement and app retention. As previous studies on player motivation suggest, intrinsic and extrinsic motivators can differently influence the way people interact with game-like systems [47,48]. Thus, user experience created by gamification is likely to differ [49].

### Limitations

The project was implemented in an uncontrolled clinical trial involving a small number of patients, which must be taken into account in interpreting the results. The usage patterns observed at posttreatment may not reflect realistic usage patterns, as the patients who already have iPhones were not able to use the system on their own smartphones.

## Abbreviations

BCBT: brief cognitive behavioral therapy
CBT: cognitive behavioral therapy
EMI: ecological momentary intervention
mHealth: mobile health
SDK: software development kit
UCD: user-centered design
